# Consciousness at sea

**DOI:** 10.1098/rstb.2024.0313

**Published:** 2025-11-13

**Authors:** Irina Mikhalevich

**Affiliations:** ^1^ Philosophy and Religious Studies, North Carolina State University at Raleigh, Raleigh, NC 27607, USA

**Keywords:** adaptationism, evolutionary progressivism, trait individuation, consciousness

## Abstract

This essay articulates three distinct but interrelated challenges facing evolutionary explanations of consciousness. These are: (i) lingering misconceptions about evolutionary explanations that stem from evolutionary progressivism and adaptationism; (ii) the ‘measurement problem,’ or the challenge of gathering comparative data on consciousness, as required by any evolutionary accounts, in the absence of not just a theory of consciousness but uncontroversial meta-strategies for coping without such theory; and (iii) unresolved bio-theoretical challenges about how best to individuate traits for the purpose of functional analysis. The effects of these challenges are then illustrated by looking at adaptive accounts of consciousness that pick out valence (provisionally, the goodness or badness of psychological states) rather than phenomenal consciousness (p-consciousness, or the ‘something it is like of experience’) as the fitness-conferring character. On these accounts, p-consciousness is adaptive only if valence and p-consciousness are parts of the same trait; otherwise, p-consciousness appears to be a byproduct of selection for valence. Whether p-consciousness and valence are part of the same trait in turn depends on how traits are individuated and the relationships between valence and p-consciousness.

This article is part of the theme issue ‘Evolutionary functions of consciousness’.

We are like sailors who must rebuild their ship on the open sea, never able to dismantle it in dry-dock and to reconstruct it there out of the best components. Where a beam is taken away a new one must at once be put there, and for this the rest of the ship is used as support. In this way, by using the old beams and driftwood the ship can be shaped entirely anew, but only by gradual reconstruction.

― Otto Neurath [1]

## 1. Introduction

Few research domains are as epistemically contentious and morally significant as the study of consciousness. Within ethics, consciousness is widely held to mark the difference between those who have moral standing (those who matter morally in their own right) and those whose who lack it—viz., those who matter only instrumentally or derivatively, if at all. Clarifying the nature of consciousness could thus not only improve our understanding of a highly prized feature of human experience but also reveal the distribution of morally valuable life. The ethical and public policy implications extend beyond questionably conscious humans (e.g. early-stage fetuses, people suffering from disorders of consciousness) to all biological organisms—and beyond to artificial intelligence, brain organoids and artificial forms of life. And yet, not only is there little agreement about what consciousness is, but also about what a satisfactory theory of consciousness might look like or how to approach the matter empirically. And while there is broad agreement that consciousness is an evolved biological trait, there has been little systematic investigation into what an evolutionary account of consciousness must look like or how it is to handle these existing conceptual and methodological disagreements. If Neurath’s famous metaphor of scientific knowledge as the process of repairing a ship mid-voyage was ever fitting, it is here, with the naturalistic approach to consciousness. In what follows, I will venture to describe some of the perils of this journey and to pitch in with my shipmates to shore up the evolutionary planks of this vessel.

Less metaphorically: we begin in §2 with a very brief history of naturalistic approaches to consciousness. Section 3 articulates three distinct but interrelated methodological and conceptual challenges facing evolutionary explanations of consciousness. These include: (i) lingering misconceptions about evolutionary explanations, most notably the idea that evolutionary transitions should be glossed as progressive and that among evolutionary explanations, only adaptive ones are properly explanatory; (ii) the ‘measurement problem’, or the challenge of gathering comparative (animal) data on consciousness, as required by any evolutionary accounts, in the absence of not just a theory of consciousness but uncontroversial meta-strategies for coping without such theory; and (iii) unresolved bio-theoretical challenges about how best to individuate traits for the purpose of functional analysis. Section 4 presents a case study that illustrates how these challenges show up in arguments favouring adaptive explanations of consciousness, focusing on adaptive accounts that pick out valence (provisionally, the goodness or badness of psychological states) rather than phenomenal consciousness (or the ‘something-it-is-like’ of experience; henceforth just ‘consciousness’ [2]) as the fitness-conferring character. On these accounts, it will be suggested, consciousness is adaptive only if valence and consciousness are parts of the same trait; otherwise, consciousness appears to be a byproduct of selection for valence. And whether consciousness and valence belong to the same trait in turn depends on: (i) how traits are individuated and (ii) the relationships between valence and consciousness.

## 2. From the armchair to the laboratory

Not long ago, the mind sciences viewed the very idea of ‘consciousness science’ as a misnomer. Consciousness appeared to be unobservable and ill-defined and thus the proper domain of armchair philosophy, not amenable to empirical investigation. The cognitive revolution that displaced behaviourism in the latter half of the 20^th^ Century gave license to investigate the inner workings of mind, but these were confined to cognitive mechanisms and their neural underpinnings—not consciousness. Moreover, the cognitive revolution did not reach comparative (animal) cognition until some decades later, thus delaying the introduction of valuable comparative data that might have dislodged anthropocentric assumptions in human psychology before they could take root. Indeed, that humans were conscious was rarely in question even at the height of behaviourism; consciousness was merely considered the wrong *subject*, a relic of the introspective methodologies of psychology’s early adolescence. By contrast, the comparative cognition sciences (comparative psychology, ethology) effectively denied consciousness to animals, equating the belief in consciousness with inadmissible Cartesian dualism.^1^

Even after the cognitive revolution reached comparative cognition, with both Anglophone comparative psychologists and German-speaking ethologists finally accepting the language of cognition, suspicion remained in both quarters about animal *consciousness*.^2^ As the science of consciousness was gaining ground in human psychology, the behavioural biologist Donald Griffin (1915−2003) struggled to convince fellow ethologists to develop what he termed a ‘cognitive ethology,’ which took seriously the inner subjective worlds of animals [3,4]. The study of animal consciousness thus lagged human consciousness studies just as animal cognition lagged human cognition. This is not to say that no progress was made at this time; only that it was indirect, tracking adjacent features, such as affect (see e.g. [5]). In short, the early naturalistic approaches to consciousness primarily sought to understand human consciousness and its evolutionary origins, and, where they ventured beyond the human case, they typically used human consciousness as a point of departure. Yet because the science of nonhuman consciousness had been deferred, a properly comparative and thus properly evolutionary account of consciousness—including human consciousness—lacked the resources that it needed to accomplish its goals. Instead, these earlier naturalistic approaches homed in on the function of consciousness, its neural correlates in humans and homologous regions in nonhumans, and the likely impact on the behaviour of conscious organisms. This anthropocentric focus was a reasonable starting point insofar as introspection and verbal report were only available in our species, but it frustrated efforts to gather reliable comparative data by blurring the line between necessary features of consciousness and the contingent dimensions of human experience.

Here we should pause for clarification. The term ‘function’ is polysemous, and it is important to distinguish between two broad senses: *proximate* (mechanistic, causal) function and *ultimate* (adaptive) function [6]. Function in the proximate sense refers to the *causal role* of consciousness in the cognitive economy and its contributions to behaviour. To ask whether consciousness has a function in this sense is to ask about what it does for the organism during its lifetime. Function in the ultimate sense refers to the contributions something makes to fitness. To ask whether consciousness has a function in this sense is to ask *whether* consciousness served an adaptive function at some point in the evolutionary history of conscious beings—that is, whether it evolved because it enhanced fitness. The two senses of function are importantly related. Proximate functions help to specify mechanisms in extant organisms that can in turn suggest possible fitness advantages of consciousness (e.g. expanded behavioural repertoire, rapid learning, etc.) as well as its possible costs (e.g. energetic burden) and constraints, that may figure in assessments of adaptive function. Ultimate-cause explanations, in turn, provide the unifying theoretical framework without which we have only descriptions at the level of proximate function and mechanism. Without a deeper engagement with evolutionary theory, naturalistic approaches to consciousness remained incomplete, able to describe (and occasionally explain) only species-specific particulars, but not broader patterns in nature. Evolutionary accounts thus require comparative data.

Recent years have seen explicit and robust efforts among philosophers to integrate the cognitive science of consciousness with the biological sciences, especially comparative cognition [7,8], and evolutionary biology [9–13]. This *new naturalism*, as we might call it, is a biologically grounded approach that explicitly rejects the anthropocentrism of early naturalistic efforts in favour of inquiring about the distribution and deep origins of consciousness across the tree of life.^3^ Yet comparatively little has been said about how a properly evolutionary science of consciousness should engage with bio-theoretical questions in evolutionary theory: questions about what an evolutionary explanation should look like or how to individuate mental traits for the purposes of such explanations. The following section attempts to make progress on these questions by considering three challenges facing the new naturalistic project.

## 3. Three challenges

The new naturalism faces at least three broad conceptual and methodological challenges: The *first* stems from lingering misconceptions about the nature of evolutionary explanations, including the assumption that only adaptive explanations are properly explanatory and that mental evolution can be glossed in progressivist terms, which results in prioritizing homology over functional analogy. The *second* stems from the contested nature of the explanandum, which makes consciousness difficult to operationalize and measure. The *third* concerns strategies for approaching trait delineation both generally (for any trait) and with respect to consciousness specifically. These three problems, as we will see, are importantly interrelated: how we specify the trait of consciousness matters to whether it is an adaptation, an exaptation, a byproduct or an evolutionary accident.

### (a) Ghosts of progressivism and adaptationism past

The first challenge for the new naturalism stems from lingering misconceptions about the nature of evolutionary explanations and evolutionary mechanisms that persist in the mind sciences and stand in the way of evolutionary accounts of consciousness. Two examples are *evolutionary progressivism*, or the idea that evolution improves upon earlier forms or marches toward some sort of global optimality, such as ‘complexity, adaptiveness, self-organization, organismic autonomy or some other quantity’ ([16]; see also [17]; and *adaptationism* [18,19], or the idea that natural selection is the primary driver of evolution and that only adaptive explanations are genuinely explanatory. While few scientists today accept cruder versions of progressivism or adaptationism, echoes of both can be heard in the mind sciences generally and as concerns the evolution of consciousness specifically. While these echoes are faint and fading, they continue to exert influence and are thus worth a brief remark.^4^

Consider a subtle example of progressivism. Ginsburg & Jablonka [20] frame the emergence of putatively more sophisticated forms of consciousness in terms of major evolutionary transitions in the technical sense of Szathmáry & Maynard Smith [21].^5^ In brief, they argue that unlimited associative learning (UAL) reliably predicts a cluster of traits that jointly serve as markers of primary consciousness and that the transition from non-conscious to conscious organisms constitutes a major evolutionary transition. The later emergence of ‘imaginative consciousness’ (i.e. the ability to reflect on phenomena not present in perception) and human ‘reflective consciousness’ (i.e. symbolic, language-mediated cognition) represents additional major transitions. However, while these events may have been monumental from the standpoint of both biology and morality, and though they may be of great personal significance to human beings, they do not fit the major transitions model.^6^ And if the model is inapplicable, then all we are left with is a progressivist story privileging human minds.

To see how latent progressivism may hamper the mind sciences, consider how it delayed recognition of sophisticated cognition in birds. Jarvis *et al*. [22, pp. 151−152] write that ‘early comparative neurobiologists combined Darwin’s concept of ‘evolution’ with the nineteenth-century version of Aristotle’s ‘scala naturae’, which resulted in the view that evolution [including brain evolution] was progressive and unilinear’ such that ‘telencephalic evolution occurred in progressive stages of increasing complexity and size, culminating with the human cerebrum’ . This progressivist evolutionary picture was then encoded in classical avian brain classification, leading to the longstanding belief that avian brains were too small and structurally disanalogous to support sophisticated cognition and consciousness. The problem may have been compounded by the more subtly progressivist assumption that unseating such views required findings of structural homology with the mammalian cortex. Behavioural evidence from corvids and parrots and ‘extensive revision of our understanding of telencephalic evolution’ [22] eventually led to reclassifying the avian nidopallium caudolaterale (NCL) as *analogous* to the mammalian prefrontal cortex [23,24] . The reclassification sparked new research questions: how do tiny flight-constrained brains enable complex cognition?^7^ If convergent brain structures sustain complex cognition, could they also support consciousness [1]?^8^ Do bird brains follow uniform evolutionary trajectories, and what would divergence mean for general models of brain evolution?^9^ Abandoning progressivism shifted avian neuroscience from seeking structural homologies with human brains toward identifying functional analogies supported by lower-level (cellular, molecular, genetic) homologies. It also prompted a move from models of brain evolution as gradual accumulation towards models recognizing substantial plasticity [30,31], capable of accounting for consciousness in organisms as distantly related as octopuses and honeybees.

Next, consider adaptationism. The priority placed on adaptive explanations goes back to Darwin’s time, with Darwin’s protegee, J. G. Romanes, writing:

It is, then, adaptive action by a living organism in cases where the inherited machinery of the nervous system does not furnish data for our prevision of what the adaptive action must necessarily be—it is only in such cases that we recognize the element of mind. In other words … the distinctive element of mind is consciousness, the test of consciousness is the presence of choice, and the evidence of choice is the antecedent uncertainty of adjustive action between two or more alternatives. [32]

Similarly, Humphrey [33] opens his essay in a volume about the evolution of consciousness with the following proclamation:

Our default assumption, I believe, can and should be that living things are designed the way they are because this kind of design is—or has been in the past—biologically advantageous. And this will be so across the whole of nature, even when we come to ask deep questions about the way the human mind works, and even when what’s at issue are the central facts of consciousness. [33].

And it persists today, with Ginsburg & Jablonka [20] arguing that each transition in the sophistication of consciousness was adaptive [34].

Adaptive explanations are not the only kind of evolutionary explanation, of course, since natural selection is not the only mechanism driving evolutionary change.^10^ Thus, features may be *byproducts* of selection for something else or frozen accidents that neither serve a function nor are linked to those that do. They may also be *exaptations*: features that have been co-opted to serve an adaptive function for which they were not originally selected (e.g. feathers may have been initially selected for thermoregulation but later co-opted for flight). Adaptationism, however, either ignores non-functional possibilities or regards them as not genuinely explanatory. The view that byproduct explanations are explanatorily inferior to adaptive explanations is widespread (see [36] for discussion) but highly controversial. As Lloyd [37] argues, far from being ‘null’ hypotheses of no effect, byproduct explanations require detailed reconstruction and interpretation of causally complex developmental mechanisms that link the byproduct to the adaptive trait. And insofar as they require both an adaptive explanation and a related developmental one, they are arguably more demanding than adaptative explanations [38].

Like progressivism, adaptationism comes in more and less sophisticated forms. Since the publication of Gould and Lewentin’s *Spandrels* [18], ‘[p]hilosophers have distinguished three main forms of adaptationism: methodological, explanatory, and empirical,’ where ‘[m]ethodological adaptationism is the thesis that the best method for understanding traits is to seek adaptive accounts of their evolution… explanatory adaptationism … assert[s] that the study of adaptations is the central goal of evolutionary biology’ and ‘[e]mpirical adaptationism … is a thesis about the strength of natural selection in evolution’ [39, p. 1235]. Whether consciousness science should adopt something like methodological adaptationism is an open question, but one worth asking explicitly. Still, no matter which (if any) view of adaptationism we adopt, the question ought to be not ‘what is the adaptive function of consciousness?’ but ‘how did consciousness evolve?’

There are, however, times when we may have *prima facie* reasons to suspect that a trait is an adaptation. For example, traits that are highly complex and costly to develop and maintain seem likely to serve important functions, since they would be unlikely to arise and be retained without incentive. In such cases, we may suppose that the trait is an adaptation even when its function if we do not know *what* it does for the organism. While consciousness may indeed be complex or costly (e.g. due to being energetically demanding or requiring a tradeoff with another valuable function), these claims require independent evidence that we do not yet have.^11^ We may thus lack even this basic *prima facie* reason to treat consciousness as an adaptation. Indeed, the notion that consciousness is complex may be a holdover of the progressivist assumption that human consciousness must be sophisticated, which mistakes the subjective value of our own experience for the objective measure of complexity.

In summary: evolutionary progressivism prioritizes evidence of homology with human neurocognitive systems, while adaptationism closes off explanatory avenues that ought to remain open under the new naturalistic approach to consciousness.

### (b) Conceptual development and the measurement problem

Perhaps the most pressing problem for the new naturalism is the oft-lamented fact that while consciousness is now a proper subject of scientific study, there is still significant disagreement about what it is—including whether it exists at all—and how to study it in nonhuman systems. The apparent intractability of the consciousness question has generated meta-level discussions about why these problems appear intractable, whether they are, and how consciousness science ought to proceed if they remain intractable (see [40]). In a recent review paper, Seth & Bayne [41] identify dozens of theories of consciousness (ToCs), each with its own empirical implications. As they write: ‘[f]or the most part, ToCs have tended to focus on particular kinds of local states (perceptual experiences, with an emphasis on vision), on particular kinds of global states (ordinary waking awareness) and on particular kinds of conscious creatures (adult human beings)’ [41, p. 449]. In other words, the empirical basis of the theoretical projects are themselves arguably inadequate for a broad theory of consciousness: one that extends to all conscious *states* and all conscious *creatures*. Thus, the first problem facing the new naturalism in consciousness science is the old problem of how to study (observe, test and measure) something that we are uncertain to exist and that we cannot seem to define.

The problem of the ambiguous explanandum and the subsequent need to calibrate among theoretical concepts and measurement practices may be particularly pronounced in the case of consciousness, but they are not unique to consciousness studies. All sciences with elusive target phenomena face this problem to some degree, especially in the early stages when concepts have yet to be fixed. Take the nearby example of mindreading, or mental-state attribution, in nonhuman animals. Not knowing precisely how mindreading presents across all species ‘makes it difficult to provide direct support for a [mindreading] hypothesis … because researchers are not certain what pattern in the data one is entitled to expect under the supposition that the hypothesis is true’ [42, p. 37]. In the history and philosophy of measurement and experiment, this is the challenge of the co-stabilization of theoretical concepts and measurement practices [43,44]. Experimental measurements of a loosely specified target phenomenon contribute to the refinement of the concept that describes the phenomenon, which in turn helps refine and direct future measurement practices, and so on. Seth [12,41] is confident that this ‘measurement problem’ can be overcome in the same way that similar problems were overcome in the physical sciences, such as the development of the concept of temperature and the instruments used to measure and quantify it (see [45] for historical discussion).

How can consciousness science proceed when there is so little agreement about the explanandum and when we lack not just all the relevant data that might resolve the issue, but also confidence in the measurement instruments that we use to gather the data? Some evolutionary approaches that take the ‘creature feature’ of Seth *et al*.’s critique seriously by testing for consciousness among nonhuman organisms and entities adopt what Birch [46] calls the ‘theory-heavy’ approach of presupposing a specific account of consciousness. This strategy, however, risks either eliminating nonhumans by definitional fiat (e.g. by assuming a theory of consciousness that makes high cognitive demands, such as requiring language, or one that links consciousness to mammalian cortical structures) or including them prior to gathering empirical evidence (e.g. on the information integration theory (IIT) of consciousness). Other approaches attempt to sidestep the theoretical debate altogether by proceeding directly to the evidence-gathering stage without first offering a theory of the mechanisms responsible for consciousness, typically by proposing that certain features signal the presence of consciousness. Ginsburg and Jablonka’s UAL [20], for instance, is one proposed ‘marker for the evolutionary origins of consciousness’ [47]. The obvious problem with such ‘theory-neutral’ approaches is that absent a theoretical basis, there is little reason to suppose that features like UAL are reliable markers of consciousness [47]. Birch [46] thus proposes a ‘theory lite’ approach that identifies likely markers of consciousness based on their theorized contributions to elements of conscious experience. The ‘theory’ in ‘theory lite’ approaches is thus not a general theory of consciousness, but a theory about how specific behavioural, neurobiological and cognitive features probably contribute to conscious experience. Notably, the list of markers on Birch’s approach is provisional and revisable. In the same spirit, Andrews recommends a dynamic marker approach (DMA) to animal consciousness, ‘which starts by identifying the properties that trigger human commitments to consciousness in familiar animals and then derives further markers that can be used to identify consciousness in unfamiliar ones’ [48]. By facilitating empirical exploration, Andrews hopes that the DMA and similar ‘theory-lite’ approaches could aid in the eventual development of a well-evidenced theory of consciousness that might then be deployed in the service of further data collection until an equilibrium is reached between theory and data. Meanwhile, Seth & Bayne [41] embrace a neo-Popperian strategy that encourages the parallel development and subsequent severe testing of *competing* ToCs in the hopes that only the most plausible ones will survive. They articulate several criteria that ToCs must satisfy to be properly testable against one another and the world, including the minimal requirement that ToCs clearly articulate their theoretical commitments and empirical implications and reject the anthropocentric focus on humans as the paradigm of consciousness. If ToCs can meet these requirements, they optimistically write: ‘there is every reason to think that the iterative development, testing and comparison of ToCs will lead to a much deeper understanding of this most profound of mysteries’ [41, p. 449].

What can evolutionary accounts of consciousness do in the meantime? Ironically, if we knew the adaptive function of consciousness, we would know which selective pressures tend to give rise to it and could then test for consciousness among lineages that have faced similar pressures, thereby gathering new comparative data needed to resolve disputes among ToCs. However, we cannot determine the functional profile of consciousness without the comparative data, which in turn requires a catalogue of reliable consciousness markers. The new naturalist approach seems to require a more elaborate sort of reflective equilibrium than what either Andrews or Seth [41,48] imagine, since in addition to ToCs and the empirical evidence from comparative, neuroscientific and psychological research, we need to add evolutionary theory. As we have already seen, the question of what makes for an evolutionary explanation is not settled, and as we will see next, neither is the question of how traits—such as consciousness—are to be individuated.

### (c) Trait individuation and consciousness

The trait-identification problem arises from the challenge of (metaphorically) carving continuous, causally integrated, complex organisms at their conceptual joints. In a recent paper, DiFrisco & Ramsey [39] write that ‘whether a trait is considered an adaptation crucially depends on how the trait is individuated as well as how significant a role selection played in its history.’ This is not the trivial point about the need to clearly articulate an explanandum: the trait-individuation problem is both under-appreciated and remains largely unresolved within theoretical evolutionary biology and it has rarely been explicitly addressed in cognitive science (see [49] for an exception that proves the rule). On some accounts, consciousness may turn out to be an adaptation; on others it may be a byproduct; while on others there may not be any mind-independent fact of the matter.

Broadly speaking, *traits* can be glossed as ‘heritable features that are the same across species despite much interspecies and intraspecies variation’ [49]. The literature appears to be divided among accounts that individuate traits along (i) their structural or phenomenological features; (ii) their adaptive functions (selectionist approaches); and (iii) those that treat trait identification as mostly or entirely arbitrary or determined by specific research goals (pluralistic approaches) [39]. To individuate traits phenomenologically is to classify them according to their observable features (e.g. a forelimb; an eye). However, phenomenological approaches offer no guidance about which features of a continuous organism may be singled out as traits apart from their subjective appearance to human beings. At best, phenomenology might guide provisional classifications that subsequent analysis might revise; at worst, it is arbitrary and thus unscientific. In either case, we may set it aside. Pluralistic approaches recognize that different research programmes will use classification schemas that serve unique ends: e.g. phylogenetic reconstruction may call for an approach that emphasizes homology (and thus relatedness) while macroevolutionists may be more interested in identifying broad patterns in nature and thus focus on homoplasies. For the selectionist, features of an organism comprise a single complex *trait* (e.g. an eye, a wing, and heart) if they were selected to serve the same adaptive function (e.g. vision, flight, perfusion of oxygenated blood). Traits, on this view, are thus those complexes of an organism that are visible to selection, and all and only adaptations are traits. On this approach, consciousness would not count as a trait without a history of selection for its current function.

No matter which approach one takes, defining a trait too narrowly risks defining not the evolved trait, but a specific instantiation of it within a given species. To use a well-worn example, to define *flight* in terms of the specific biomechanics of avian bodies is to conclude (absurdly) that bats and bees do not fly. As Figdor [49] writes with respect to cognition, but equally applicably for consciousness, ‘[i]f we accept that cognition evolved, then we ought to define cognitive types that are not *a priori* uniquely human.’ This is because, as Figdor rightly notes, traits are *multiply realizable* historical (aetiological) kinds that are often arrived at convergently. She thus critiques a standard anthropocentric approach to cognitive trait analysis that begins by taking the human case as exemplar and seeking out homologous rudiments of that cognitive ability elsewhere. The anthropocentric strategy, in other words, picks out features of the human phenotype and illicitly treats them as the trait. To avoid this mistake, Figdor recommends broadening the evidence base to other organisms in the process of homing in on the cognitive trait—a recommendation that applies to consciousness as well. And this requires taking care to avoid building in any proximate mechanisms, such as specific neural substrates, to the working definition of consciousness [50].

Yet, while differences between traits and their physical realization base are relatively clear for traits like *wings*, they are less obvious for mental state terms like consciousness. First, some accounts of consciousness define it not *in connection with*, but at least partially *in terms of* its physical structures and processes. By contrast, *wing* is defined functionally rather than biomechanically. The proximate function of consciousness, however, is disputed. Second, what counts as a proximate mechanism for consciousness—the sort of mechanism that belongs to the phenotype sense but not the character sense of function—is unclear. For example, both architectural features of brains (structures such as the cortex or thalamus) and processes (such as whole brain activation and sleep/wake cycles) have been implicated in consciousness such that both might be rendered as proximate mechanisms. Yet while including cortico-thalamic structures and their homologues in the specification of a consciousness trait seems to straightforwardly collapse contingent expressions of the trait with the character of that trait, the same is not true of processes such as patterns of activation because processes are in principle multiply realizable. Insofar as activation patterns appear to be robust across phyla, they may be not only useful markers, but even themselves be *parts of* the trait (see [13,51]).^12^ Yet, these patterns may not be robust across all conscious organisms, and we certainly have no reason to expect them to be in organisms and entities that did not evolve on Earth. If consciousness is multiply realizable across both structure and patterns of activation, then it would be a mistake to include specific processes in the definition of the trait. Even less clear, however, is whether consciousness should be defined in terms of other psychological and behavioural phenomena, such as cognition, action or affect. In short, what *counts* as a proximate mechanism of the sort that should be excluded from the definition of a trait remains an open question.

Put another way: individuating the trait of consciousness requires clarifying which features of related (psychological, neural and behavioural) phenomena are proper parts of consciousness and which are merely contingently related. The next section shows that several adaptive accounts of consciousness lean on features associated with consciousness, like valence, without clarifying how these features are related to consciousness. The result is that such accounts fail to establish that consciousness itself is an adaptation.

## 4. Case study: adaptive accounts of consciousness and valence

### (a) Adaptive accounts of consciousness

Differences among accounts of the evolutionary origins of consciousness reflect the differences among ToCs. Certain higher-order theories (HOTs), for instance,^13^ suggest that consciousness evolved recently, either with human language or with the sophisticated cognitive gadgets that permit human-style thinking [53]. Such accounts may be limited in the sort of evolutionary narratives they can provide, as narrative reconstructions of the one-off emergence of consciousness in a single hominin species are unlikely to support inferences about the patterns governing the evolution of consciousness more generally. On the other end of the spectrum are accounts that take consciousness to be fundamental to all life and thus deeply ancient. Whether consciousness has an adaptive function on these ‘biocentric’ accounts crucially depends on whether it plays a causal role in the organization of life. Ironically, these expansive accounts face the same ‘N of one’ problem as anthropocentric accounts since they presume consciousness to have a single origin, despite the sample itself containing all Earthly life. Most evolutionary accounts fall somewhere in between these extremes, plausibly supporting claims of convergence on consciousness and thus inferences about the conditions under which it may emerge anew.

One family of views takes consciousness to have arisen (gradually) alongside the emergence of complex body plans and the mobile behaviours that they permit. Complex moving bodies require the coordination of many parts—giving rise to the need to track the internal states of the organism (interoception)—while navigating complex environments requires appropriate sensory modalities (exteroception) and the ability to maintain a continuously updated internal representation of the organism’s bodily movements in space that reliably distinguishes between its own movements and the environment (an ‘efference copy’).^14^ Finally, the organism must be able to combine and leverage this information into adaptive action. Views differ about where in this evolutionary process consciousness arose—whether as a solution to the reafference problem [51,54]; with the capacity to learn [11]; with motivation towards adaptive action [55]; or alongside the emergence of coherent agency [13].

However, because similar origin stories can and have been told for *cognitive* function alone, adaptive accounts of consciousness need to further explain the unique contributions that consciousness makes *over and above* those of cognition. Adaptive accounts thus have the explanatory burden of showing how consciousness can be visible to selection. Since the most plausible path to visibility is through shaping behaviour, these accounts take consciousness to directly contribute to adaptive action, typically by facilitating the cognitive abilities behind action-selection [46].

Put another way, adaptive accounts must assign a proximate role to consciousness in adaptive behaviour (e.g. unification of cognitive states under centralized control, a facilitating role in action-selection, etc.). For example, the UAL theory attempts to do this by drawing on global workspace theories (GWTs), which take consciousness to be centrally involved in the coordination of parallel information-processing sub-systems in the mind/brain, thus serving as a ‘central information exchange, allowing some processors—such as sensory systems in the brain—to distribute information to the system as a whole’ [56]. According to GWTs, consciousness arises from a quasi-Darwinian competition among cognitive states in which the winners are ‘broadcast’ (made available) to the whole neural–cognitive system, with attention acting as a spotlight. If consciousness serves the function of unifying distributed information and making it available to action-selection that benefits the whole organism, then its adaptive value seems obvious. Baars *et al*. [57] write that ‘...sensory consciousness is profoundly embedded in biology, anatomy, physiology, and above all, in adaptive brain functions that serve us in every second of waking life.’ However, being *embedded in* features that contribute to adaptive action—no matter how profoundly—is far from being a contributor to them. It is unclear whether consciousness is responsible for unification or is a mere byproduct of it. Even more problematically, while the mental states that are broadcast to the system become ‘access-conscious’ (i.e. made accessible to other states), it is not clear that they also become phenomenally conscious (subjectively felt). Thus, while the information-integration, unificatory and spotlighting for action-selection functions are likely adaptive, GWTs appear to (potentially) explain the origins and proliferation of consciousness without assigning to it a functional (causal) role. Ultimately, GWTs do not distinguish among adaptive and byproduct accounts of consciousness and the UAL inherits this problem.

Next, consider adaptive theories that focus on *affective* consciousness rather than *sensory* consciousness and that lean on the role played by conscious valenced affective states in guiding organisms towards adaptive action. Veit [58] writes: ‘it is an influential view among those taking an explicitly evolutionary approach to consciousness, to see a currency of evaluation as something quite central in making sense of subjective experience in nature’ [58, p. 285]. More explicitly, Birch [59] argues that ‘experience presents an imperative or command—roughly, get more of this! [or less of this!], where this refers to the experience itself’ and the more/less distinction tracks whether the experience is positively or negatively valenced [59]. Valence, for Birch, is an imperative that is present in conscious experience. Likewise, Veit [55] maintains that ‘pleasure and pain are central in the evolution of animal life’ and that ‘valence evolved as a proximate common currency for action selection that reflects the fitness values of alternative actions.’ On this view, negative valence is coextensive with pain and positive valence with pleasure, and these are sufficiently accurate projections of the fitness values of the possible actions among which an organism must choose. Valence, thus understood, assigns subjective values to states in the world and either compels or advises the organism to select the action with the highest projected value. Vallortigara [54] similarly glosses valence as an ‘internalized evaluative response’; and Cleeremans & Tallon-Baudry [60] defend a ‘phenomenal worthiness’ hypothesis of conscious function, according to which conscious experiences embed subjective hedonic values that motivate and guide behaviour. ‘Why’, they write, ‘would we do anything at all if the doing was not doing something to us?’ On their view, it is only if values are ‘intrinsically’ (non-derivatively) embedded in subjective experience that they can offer a ‘reason’ for action. Jaak Panksepp and colleagues echo this sentiment when they describe their influential affective neuroscience research programme as ‘starting from the assumption that ‘rewards’ are rewarding because they feel good’ [61, p.189].

There is much to be said for these valence-leaning approaches to the evolution of consciousness because valence is a central feature of affective states (emotions, moods, attitudes) on all theories of affect [62] and affective states plausibly serve important roles in adaptive behaviour. At the individual level, valenced sensations (pleasures, pains, etc.) and emotions (happiness, sadness, etc.) serve as internal punishment and reward systems that may facilitate adaptive approach and avoidance behaviours and aid in learning [63]. At the group level, emotions and moods (depression, anxiety, optimism, etc.) serve critical social functions by communicating the inner states of cooperative partners, securing bonds of attachment, etc. [64].

Suppose *arguendo* that the valenced component of affect is adaptive. Does it follow that consciousness is adaptive as well? The answer will depend on whether valence and consciousness are features of the same trait, which in turn depends on: (i) the relationship between valence and consciousness; and (ii) how we determine when two features are part of the same trait. To further illustrate the interdependence of the three conceptual problems raised in §3, let us consider these questions in turn.

### (b) The relationship between valence and phenomenal consciousness

Carruthers [65] writes that although the cognitive neurosciences widely view valence as a ‘common currency’ for action-selection, valence itself has been surprisingly undertheorized. In a series of papers, he attempts to remedy this oversight, beginning with the question of whether ‘valence makes a constitutive rather than a causal contribution to phenomenal experience’ and how we might know ‘whether valence is phenomenally conscious in its own right’ [65], p. 671]. These are the very questions we must ask in determining whether action-selection accounts of the evolutionary function of consciousness succeed in showing that consciousness is adaptive.

Carruthers [65] distinguishes between non-representational ‘hedonic’ accounts and representational accounts on which valence is ‘nonconceptual representational content’. On the former, valence is ‘a distinct qualitative property that [affective] experiences possess, where this property is … regarded as intrinsically [good/bad]’ [65], p. 664]. On the latter, ‘valence is an intrinsically motivating nonconceptual representation of goodness or badness’ or ‘value’ [65], p. 661]. This content is ‘fine-grained’ and ‘perception-like,’, though distinct from the sensations given in conscious experience. In a later paper (2023), he glosses the nonconceptual content account as a form of *evaluativism*, which takes valences to be ‘analogue-magnitude representations of value’ [63, p. 534] that ‘reliably [carry] information about the adaptive value of items or events in the environment or body’ [63, p. 540] for the organism. The alternative *imperativist* accounts regard valences as ‘graded-strength imperatives with the content, ‘more of this!’ and ‘less of this!’’ (see [59,63, p. 534] above) or ‘more/less of this *for me*!’. While both approaches take valence to be motivating, only imperativism takes it to be *directly* motivating. On his representationalism/evaluativism, valences interact with downstream cognitive and attentional systems to promote decision-making and learning and may contribute to consciousness.^15^

Carruthers [63] defends the representational/evaluativist views as (inter alia) the more parsimonious option. He argues that imperativists fail to appreciate that in addition to imperative content, valences must also involve *representations* of the adaptive value of states of affairs, since ‘it is the presence of th[e] information [contained in these representations] that explains how the role of valence has become stabilized in decision making and evaluative learning’ [63, p. 540]. By contrast, evaluativism assigns only representational content to valence. However, simpler explanations are not thereby more likely to be true because the world may not itself be simple and because *simplicity* is surprisingly difficult to define and operationalize—particularly with respect to the mind [66–68].^16^

The aim here is not to settle the imperativism/hedonism versus evaluativism/representationalism debate nor even to present it in full, but to consider the implications of each view for the relationship between valence and consciousness for the purposes of assessing the evolutionary status of consciousness. On this score, Carruthers writes that ‘[w]hile both accounts of valence can justify the claim that valence *can be* phenomenally conscious, the hedonic account (*and only the hedonic account*) seems to entail that valence is *always* phenomenally conscious’ whereas ‘there need be nothing intrinsically conscious about nonconceptual representations of value’ ([65], p. 673], emphasis added). In other words, if hedonic accounts of valence are right, then valence and consciousness are constitutively related; if evaluativist–representational accounts are right, then they are causally related and can come apart. And, if imperativism implies motivational hedonism, as Carruthers maintains, then imperativism also entails that valence is necessarily conscious. This means that if the relationship is constitutive, then the distribution of *consciousness* will exactly track that of valence.

Comparative data showing how valence is implemented in distantly related organisms could shed some light on the evolvability of (affective) consciousness on either account of valence, but their implications would only be straightforward for hedonism/imperativism.^17^ For example, Feinberg and Mallatt [14] describe what Gillette and Brown [71] call the ‘sensory integrator circuit for incentives’ in gastropod molluscs, which ‘labels the sensory input it receives as either rewarding (+) or aversive (−) then … codes the motivations that dictate approach (+) versus avoidance (−) behaviours’ and is associated with behavioural controls and ‘potentiates *memories* of the salient stimuli, for associative learning’ ([14, p. 122]; emphasis in original). However, they speculate that despite representing values, ‘this incentive circuit may have too few neurons to produce true, affective, experiences’—that is, consciousness (ibid). Godfrey-Smith [10] similarly writes that there ‘seems no reason to think that evaluations must always be felt’ since even the presumably non-conscious ‘bacteria and unicellular organisms show patterns of approach and withdrawal’ indicative of evaluative ability [10, p. 13]. He suggests that ‘evaluation give[s] rise to genuine [conscious] feeling’ only with instrumental learning, glossed as the ‘capacity to open-endedly associate specific behaviors … with an evaluation of their effects’ (ibid). The incentive circuit in gastropods thus seems to fit Godfrey-Smith’s criterion for consciousness. Feinberg and Mallatt [14] and Godfrey-Smith [10] seem to assume a *causal* relationship between consciousness and valence, but with different results. If constitutive views are right, however, then gastropods are phenomenally conscious regardless of their capacity for associative learning or the complexity of their evaluative circuitry. And if approach/avoidance behaviours reliably indicate valence, then constitutive accounts may even imply consciousness in organisms as ‘simple’ as bacteria.^18^

This may seem like a problem for constitutive accounts. Indeed, unconscious representations of value seem common (e.g. in AI systems that assign utility values to action alternatives). Yet, it is one thing to imagine unconscious value in AI and bacteria and another to show that it can play a motivational role in more ‘complex’ organisms. Echoing this scepticism, Mahr & Fischer [72] speculate that ‘the [conscious] experience of valence might allow one to learn value information that its purely propositional [i.e., non-conscious] representation could not.’ And if consciousness carries important information (or serves to integrate or otherwise facilitate such information), then it may be required for complex decision-making, whereas simpler lifeways could make do with representation alone. Let us then (very briefly) consider evidence for unconscious representation of value in humans and other mammals.

Psychological studies and everyday experience suggest that people regularly experience moods and emotions without realizing it, even as these states influence their judgement and behaviour [73]. This possibility suggests a three-way dissociation among valence, consciousness and behaviour—contra hedonism and imperativism. Consistent with this reading, Carruthers [63] writes that while imperativism treats valence as ‘essentially a high-level motor instruction … action tendencies are produced directly and automatically, and independently of the valence component of affective states [in human brains]’, suggesting that ‘the valence component and the action tendencies have a common cause … rather than either one proceeding via the other’ [63, p. 536]. However, there is some dispute over how to interpret the behavioural evidence—e.g. whether it is the emotion itself that is unconscious or merely its cognitive content and broader significance [74,75]. After all, failing to *recognize* what one feels is not failing to *feel* anything [76]. As Arnaud [73] puts it, ‘emotions always color consciousness even when we are not conscious of them.’ Neither is the evidence from affective neuroscience uncontroversial, as the relative contributions of subcortical areas to affectivity in humans and other animals remain contested [53,61]. Note, however, that even if valence and consciousness *can* decouple in humans without significant loss of function—thus lending support to evaluativism—we should not overinterpret this evidence to mean that it *must* be unconscious in other normally functioning organisms.^19^

In summary: we might provisionally conclude that *if* valence is representational (if evaluativism is true), then its relationship to consciousness is *causal*, whereas if valence is hedonic (if imperativism is true), then the relationship is *constitutive*. Which view is right depends, in turn, on a number of unresolved questions about the phylogenetic distribution of valence and the implementational requirements for valenced behaviour. The next section examines how *causal* and *constitutive* views of the valence/consciousness relationship inform two key questions: whether valence and consciousness constitute a unified trait, and on which of these accounts consciousness may be an adaptation.

### (c) Trait individuation and consciousness

Let us now put the causal/constitutive discussion together with the trait-individuation issue and ask what valence-leaning accounts must do to establish consciousness as an adaptation rather than byproduct of selection for valence. In the interest of space, let us assume a selectionist approach to trait-individuation.

#### (i) Is consciousness an adaptation if the relationship is constitutive?

If valence and consciousness are *constitutively* related, then either they are (i) parts or properties of the same phenomenon (as *bones* and *feathers* are parts of a bird wing, and *colour* and *shape* are its properties); or (ii) one is a part or feature of the other.

It is not necessary to examine each permutation to notice that whether consciousness is an adaptation or a byproduct depends on the nature of the constitutive relationship. This is because the mere fact that some feature, F, always bears some property, *p*, says nothing about whether F and *p* are part of a single trait on anything but the phenomenological account of trait-identification. To illustrate, suppose that all bird feathers were blue owing to some quirky developmental constraints without this playing any role in the function of *flight*. Even if blueness were a property of all feathers, it would not be part of the trait of a *wing*. A simple counterfactual reveals why: if the feathers were a different colour, the wings’ function would remain unchanged. The counterfactual exercise allows us to sift necessary properties of a trait (those that contribute to a common function) from contingent ones (those that do not). Unlike certain structural characteristics, such as the hollowness of feathers, colour is a contingent property of bird wings relative to the function of flight (though not perhaps to the functions of camouflage or mate selection). Thus, merely showing that consciousness is a property of valence for all organisms on Earth would not establish it as an adaptation. Hedonic/imperativist accounts must further show that consciousness is not just a *contingently* constitutive feature (like feather colour), but one that was selected for its contribution to the function played by valence. Failing to do so fails to differentiate between byproduct and adaptive accounts.

#### (ii) Is consciousness an adaptation if the relationship is causal?

Suppose representationalism/evaluativism is true and that the relationship is instead causal. This might be because (inter alia): (i) consciousness and valence are products of a common cause, such as a single neuro-cognitive mechanism; (ii) valence causes consciousness; (iii) consciousness causes valence; (iv) they independently contribute to a common process; or (v) they are elements of a complex, dynamic system with several causal pathways connecting them.^20^ Option (ii) seems to render consciousness a byproduct of selection for valence, while option (iii) treats consciousness as an adaptation insofar as it potentiates the functions that valence produces. However, since valence is glossed as the representation of value on the representational/evaluativist account, it is difficult to see how it could cause or be caused by phenomenal consciousness. Assuming that valence is an adaptation, under which of the remaining causal conditions could consciousness and valence constitute parts of a single adaptive trait?

The selectionist answer will depend on whether both consciousness and valence were selected for a shared adaptive function (e.g. action-selection, complex decision-making, learning) either jointly or in stages. For example, consciousness might have initially emerged as a byproduct (owing to a developmental link with valence) and later been recruited (*exapted*) for complex decision-making, action-selection, etc.^21^ In this scenario, consciousness and valence would be *both* developmentally linked *and* jointly contribute to a common adaptive function. Causal account (iv) straightforwardly supports the joint contribution to adaptive function, while (v) permits it. Representational/evaluativist accounts must either show how option (iii) is possible or offer an account of the causal mechanisms behind (iv) or (v) before concluding that consciousness is an adaptation.

To sum up: valence-leaning accounts that aim to secure an adaptive function for consciousness must establish that consciousness and valence constitute the same adaptive trait. At first glance, constitutive accounts seem better positioned to do so insofar as they *define* valence and consciousness in terms of one another. But, as we saw, not all parts or properties of adaptive features are themselves adaptations. Likewise, without differentiating among types of causal accounts, valence-leaning accounts that adopt representationalism cannot differentiate between adaptive and byproduct accounts of consciousness.

And it is here that the methodological adaptationism question arises: how long should we continue seeking adaptative/functional explanations for a phenomenon and when it is rational to look to alternatives? Lloyd [37] defended her byproduct account of the female orgasm against both philosophers who wrongly took ‘byproduct’ to be pejorative and adaptationist scientists who wrongly viewed byproduct accounts as explanatorily vacuous and equivalent to ‘scientific surrender’. Similarly, ethicists may (wrongly) worry that byproduct accounts of consciousness may undercut its (ethical, personal) significance, while scientists and scientifically inclined philosophers may (wrongly) worry about premature surrender. But Lloyd may have missed another reason why byproduct explanations appear unsatisfying: viz, that they seem too quirky and contingent to be useful for making predictions about where to expect consciousness next. By contrast, one might expect adaptationist accounts to support inferences about law-like patterns in nature. These are not idle concerns: if byproduct explanations can equally support inferences about law-like patterns, then there is less reason to persist in seeking a function of consciousness when an equally plausible account is already to hand. If, however, byproduct accounts are in principle unable to meet this challenge, then continuing to pursue an adaptationist answer may be the rational choice. These questions will not be resolved here, but addressing them directly is preferable to allowing them to covertly shape consciousness research.

## 5. Conclusion

I have suggested that the new naturalist programme in consciousness science would do well to include discussion of bio-theoretical questions about trait identification and the desiderata of evolutionary explanations. Doing so can reveal hidden assumptions about explanatory strategies (progressivism, adaptationism), paving the way for a broader class of evolutionary explanations. Consciousness may or may not have a function; nevertheless, it evolved. The task is to figure out why.

There is, however, another task: to bring evolutionary questions to bear on questions about the possibility of consciousness beyond the familiar Earth-bound biological case. The evolutionary history of consciousness holds some promise here: if, for example, consciousness always arises under specific ecological conditions or is always co-extensive with features such as valence, then we have some (defeasible) reason to suspect that this robust regularity will obtain in other contexts as well (e.g. with artificial intelligence, artificial life or in life beyond the Earth). However, it is also important to recognize the limitations of projectability. And for this—alas—we need to steer our ship into deeper conceptual waters.

## Data Availability

This article has no additional data.

## Footnotes

^1^
Ironically, these views were motivated in part by an implicitly Cartesian mechanistic approach to animal behaviour.

^2^
This reluctance to investigate consciousness may at first blush appear to be a departure from the willingness of early comparative psychologists, such as George Jorge Romanes (1848–1894) and Conwy Lloyd Morgan (1852–1936) and even Darwin himself, to explicitly and unapologetically inquire into the evolution of consciousness in humans and other animals. However, Darwin and the early evolutionists are unlikely to have meant by ‘consciousness’ what we mean today. Today consciousness is typically parsed into phenomenal or ‘what-it-is-like’ consciousness and access consciousness, with the former being what most philosophers mean when they mention consciousness. Darwin and his contemporaries, by contrast, were interested in the evolution of mental kinds more capaciously understood. Indeed, the distinctions among consciousness, cognition, affect and so on are modern inventions that emerged close to a century after the publication of Darwin’s *On the Origin of Species*. Thus, a return to evolutionary accounts of consciousness is not a return to these projects *per se*.

^3^
Feinberg & Mallatt's neurobiological naturalism [14] *neurobiological naturalism*, which bridges the science of consciousness and evolutionary biology, is one example of this new naturalistic approach. Building on Searle’s *biological naturalism* [15], neurobiological naturalism advocates for a neuroscientific and comparative evolutionary approach that draws on multiple lines of evidence to resolve the explanatory gap(s) of consciousness.

^4^
As Okasha [16] writes, ‘Despite widespread acceptance of the point that natural selection is a brute causal mechanism that lacks foresight … the idea that evolution is in some sense directional is still very much alive.’ While directionality need not be progressive, most directional accounts of evolution are progressive rather than regressive.

^5^
Major transitions involve the introduction of (i) a new level of selection; (ii) a new individual; (iii) new levels of ‘hierarchical control’; and (iv) an increased division of labour (ibid).

^6^
Okasha [16] notes the worry that the concept of major evolutionary transitions (METs) has ‘become too broad, sometimes seeming to include any evolutionary event that an author deems “important” enough by whatever yardstick they choose.’ He advocates defining METs terms of ‘Maynard Smith and Szathmáry’s second characterization… as the evolution of a higher-level biological unit out of formerly-free living units.’ On this definition, it is difficult to see how Ginsburg and Jablonka’s forms of consciousness could fit the MET model.

^7^
Avian brains achieve more ‘cognitive power per unit mass than mammalian brains’ by shrinking neurons while increasing density, which in turn reduces inter-neuron distances for more efficient inter-neuron communication [25, p. 7255]. Similar evolutionary strategies appear in insects [26].

^8^
Emery & Clayton [27] demonstrated that corvids and apes converged on complex cognitive function, which led others to postulate that consciousness may likewise reside in the NCL ([28]; but see [29]).

^9^
For example, Gutiérrez-Ibáñez *et al.* [30] hold that while most mammals and birds increase overall cerebral neuron number, corvids, parrots and owls preferentially invest in pallial neurons.

^10^
See, for example, Richard Prum’s famous [35] argument that the mechanism of sexual selection is both powerful and irreducible to natural selection, contra adaptationism. I am grateful to an anonymous referee for highlighting this connection.

^11^
While consciousness may be complex, we are not entitled to *assume* so because we are not yet in a position to say *what* it is. Theories that view it as complex may turn out to be wrong or may pick out neural or cognitive complexities with which consciousness is merely associated. Furthermore, we may be inclined to view the phenomenon as complex because the high-stakes discussions around consciousness are themselves terribly complex. The question is thus less about whether consciousness is complex or costly, but how *likely* it is to be complex or costly: the more likely, the more reason to adopt an adaptationist approach.

^12^
Godfrey-Smith [13, p. 1665], for instance, summarizes his tentative ‘neural dynamics of subjectivity’ (NDS) account as follows: ‘The NDS approach holds that conscious experience is characteristic of systems whose nervous systems have been shaped to realize subjectivity in the service of coherent agency, and holds that the combination of network properties and large-scale dynamic patterns seen in nervous systems is important in the explanation of consciousness, whereas the vertebrate brain architecture is inessential’.

^13^
Note, however, that not all HOTs are equally cognitively demanding, and many may thus be amenable to evolutionary explanations. See Brown, Lau, & LeDoux [52] for a review and defence of higher-order approaches.

^14^
Vallortigara writes that ‘[t]he efference copy signal may enable organisms that move to discount sensory stimulation that arises from their own actions, thereby allowing them to distinguish between the sensory stimulation caused by external stimuli and that caused by their own movements’ [54].

^15^
On attentional theories of consciousness, e.g. valence might ‘influence the competition for top-down attentional resources and entry into consciousness’ [63, p. 537].

^16^
For a sophisticated defense of imperativism that may account for this objection, see Kauppinen [69].

^17^
We must proceed with caution here since evidence for valence comes mainly from the affective and cognitive neurosciences, which remain divided about how to model affective states (e.g. as discrete or continuous) and how to interpret the neurobiological and behavioural evidence ([70]; see [61] and [52]).

^18^
For example, there is some question about whether value can be operationalized as patterns of avoidance and approach, since animals do not automatically approach valued objects or avoid disvalued ones [62].

^19^
That valence *may* be unconscious in some species (humans) *some* of the time does not mean that it *must* (or likely will) be unconscious in other organisms *all* of the time. The same argument has been made repeatedly for sensory consciousness: just because human perception is *sometimes* unconscious without dramatically disrupting function does not mean that animal perception must *always* be unconscious.

^20^
These examples are non-exhaustive and non-exclusive. All features of a single organism will be causally connected in the trivial senses of belonging to the same complex whole, resulting from a single developmental process, and having a shared evolutionary history.

^21^
Of course, consciousness could not have been exapted if it were causally inert.

## References

[1] Neurath O. 1932 Protokollsätze. Erkenntnis **3**, 204–214. (10.1007/BF01886420)

[2] Nagel T. 1980 What is it like to be a bat? In The language and thought series, pp. 159–168. Cambridge, MA: Harvard University Press. (10.1093/oso/9780197752791.001.0001)

[3] Griffin DR. 1976 The question of animal awareness: evolutionary continuity of mental experience. New York, NY: Rockefeller University Press. (10.1007/BF01067044)

[4] Griffin DR. 1978 Prospects for a cognitive ethology. Behav. Brain Sci. **1**, 527–538. (10.1017/s0140525x00076524)

[5] Panksepp J. 1998 The periconscious substrates of consciousness: Affective states and the evolutionary origins of the self. J. Conscious. Stud. **5**, 566–582.

[6] Niikawa T, Miyahara K, Hamada HT, Nishida S. 2022 Functions of consciousness: conceptual clarification. Neurosci. Conscious. **2022**, niac006. (10.1093/nc/niac006) 35356269 PMC8963277

[7] Andrews K. 2024 ‘All animals are conscious’: shifting the null hypothesis in consciousness science. Mind Lang. **39**, 415–433. (10.1111/mila.12498)

[8] Birch J *et al*. 2022 How should we study animal consciousness scientifically? J. Conscious. Stud. **29**, 8–28. (10.53765/20512201.29.3.008)

[9] Allen C, Trestman M. 2017 Animal consciousness. In The Blackwell companion to consciousness (eds S Schneider, M Velmans), pp. 63–76. Hoboken, NJ: John Wiley & Sons. (10.1002/9781119132363)

[10] Godfrey-Smith P. 2019 Evolving across the explanatory gap. Phil. Theory Pract. Biol **11**, 001. (10.3998/ptpbio.16039257.0011.001)

[11] Ginsburg S, Jablonka E. 2019 The evolution of the sensitive soul: learning and the origins of consciousness. Boston, MA: MIT Press. (10.7551/mitpress/11006.001.0001)

[12] Seth A. 2021 Being you: a new science of consciousness. New York, NY: Penguin.

[13] Godfrey-Smith P. 2024 Inferring consciousness in phylogenetically distant organisms. J. Cogn. Neurosci. **36**, 1660–1666. (10.1162/jocn_a_02158) 38579258

[14] Feinberg TE, Mallatt J. 2016 The nature of primary consciousness. A new synthesis. Conscious. Cogn **43**, 113–127. (10.1016/j.concog.2016.05.009) 27262691

[15] Searle J. 1984 Minds, brains and science, the 1984 reith lectures. London: British Broadcasting Corporation.

[16] Okasha S. 2022 The major transitions in evolution—a philosophy-of-science perspective. Front. Ecol. Evol. **10**, 793824. (10.3389/fevo.2022.793824)

[17] Powell R, Mikhalevich I. 2023 Wonderful mind: convergentism and the crusade against evolutionary progress. J. Philos. Hist **7**, 77–103. (10.1163/18722636-12341490)

[18] Gould SJ, Lewontin RC. 1979 The spandrels of San Marco and the Panglossian paradigm: a critique of the adaptationist programme. Proc. R. Soc. Lond. B **205**, 581–598. (10.1098/rspb.1979.0086) 42062

[19] Orzack SH, Patrick F. 2017 Adaptationism. In The Stanford Encyclopedia of Philosophy (ed. EN Zalta). Spring. See https://plato.stanford.edu/archives/spr2017/entries/adaptationism/.

[20] Ginsburg S, Jablonka E. 2021 Evolutionary transitions in learning and cognition. Phil. Trans. R. Soc. B **376**, 20190766. (10.1098/rstb.2019.0766) 33550955 PMC7935133

[21] Szathmáry E, Maynard Smith J. 1995 The major transitions in evolution. Oxford, UK: WH Freeman Spektrum. (10.1093/oso/9780198502944.001.0001)

[22] Jarvis ED *et al*. 2005 Avian brains and a new understanding of vertebrate brain evolution. Nat. Rev. Neurosci. **6**, 151–159. (10.1038/nrn1606) 15685220 PMC2507884

[23] Reiner A. 2005 A new avian brain nomenclature: why, how and what. Brain Res. Bull. **66**, 317–331. (10.1016/j.brainresbull.2005.05.007) 16144608

[24] Reiner A *et al*. 2004 Revised nomenclature for avian telencephalon and some related brainstem nuclei. J. Comp. Neurol. **473**, 377–414. (10.1002/cne.20118) 15116397 PMC2518311

[25] Olkowicz S, Kocourek M, Lučan RK, Porteš M, Fitch WT, Herculano-Houzel S, Němec P. 2016 Birds have primate-like numbers of neurons in the forebrain. Proc. Natl Acad. Sci. USA **113**, 7255–7260. (10.1073/pnas.1517131113) 27298365 PMC4932926

[26] Chittka L, Niven J. 2009 Are bigger brains better? Curr. Biol. **19**, R995–R1008. (10.1016/j.cub.2009.08.023) 19922859

[27] Emery NJ, Clayton NS. 2004 The mentality of crows: convergent evolution of intelligence in corvids and apes. Science **306**, 1903–1907. (10.1126/science.1098410) 15591194

[28] Nieder A, Wagener L, Rinnert P. 2020 A neural correlate of sensory consciousness in a corvid bird. Science **369**, 1626–1629. (10.1126/science.abb1447) 32973028

[29] Gutfreund Y. 2024 Neuroscience of animal consciousness: still agnostic after all. Front. Psychol. **15**, 1456403. (10.3389/fpsyg.2024.1456403) 39444826 PMC11496166

[30] Gutiérrez-Ibáñez C, Němec P, Paré M, Wylie DR, Lefebvre L. 2025 How do big brains evolve? Trends Ecol. Evol. (Amst.) **40**, 554–562. (10.1016/j.tree.2025.03.008) 40251059

[31] Güntürkün O, Pusch R, Rose J. 2024 Why birds are smart. Trends Cogn. Sci. **28**, 197–209. (10.1016/j.tics.2023.11.002) 38097447 PMC10940863

[32] Romanes GJ. 1883 Animal intelligence. vol. **44**. New York, NY: D. Appleton. (10.5962/bhl.title.1046)

[33] Humphrey N. 2000 The privatization of sensation. In The evolution of cognition, pp. 241–252. Cambridge, MA: MIT. (10.1086/498477)

[34] Belardinelli S, Pievani T. 2023 What if consciousness has no function? Biosemiotics **16**, 259–267. (10.1007/s12304-023-09533-y)

[35] Prum RO. 2018 The evolution of beauty: how darwin’s forgotten theory of mate choice shapes the animal world—and us. New York, NY: Vintage Books.

[36] Robinson Z, Maley CJ, Piccinini G. 2015 Is consciousness a spandrel? J. Am. Phil. Assoc. **1**, 365–383. (10.1017/apa.2014.10)

[37] Lloyd EA. 2015 Adaptationism and the logic of research questions: how to think clearly about evolutionary causes. Biol. Theory **10**, 343–362. (10.1007/s13752-015-0214-2)

[38] Powell R. 2012 Convergent evolution and the limits of natural selection. Eur. J. Philos. Sci. **2**, 355–373. (10.1007/s13194-012-0047-9)

[39] DiFrisco J, Ramsey G. 2023 Adaptationism and trait individuation. Phil. Sci. **90**, 1234–1243. (10.1017/psa.2023.28)

[40] Chalmers DJ. 2018 The meta-problem of consciousness. J. Conscious. Stud. **25**, 6–61.

[41] Seth AK, Bayne T. 2022 Theories of consciousness. Nat. Rev. Neurosci. **23**, 439–452. (10.1038/s41583-022-00587-4) 35505255

[42] Halina M. 2024 Animal minds. Cambridge, UK: Cambridge University Press.

[43] Feest U. 2012 Exploratory experiments, concept formation, and theory construction in psychology. Sci. Concepts Invest. Pract. **3**, 167–189. (10.1515/9783110253610.167)

[44] Keyser V. 2021 Experimental effects and causal representations. Synthese **198**, 5145–5176. (10.1007/s11229-017-1633-3)

[45] McCaskey JP. 2020 History of ‘temperature’: maturation of a measurement concept. Ann. Sci. **77**, 399–444. (10.1080/00033790.2020.1817980) 32981446

[46] Birch J. 2022 The search for invertebrate consciousness. Noûs **56**, 133–153. (10.1111/nous.12351) 35321054 PMC7612530

[47] Halina M, Harrison D, Klein C. 2022 Evolutionary transition markers and the origins of consciousness. J. Conscious. Stud. **29**, 62–77. (10.53765/20512201.29.3.077)

[48] Andrews K, Monsó S. 2021 Animal cognition. In The Stanford Encyclopedia of Philosophy (ed. EN Zalta). Spring. See https://plato.stanford.edu/archives/spr2021/entries/cognition-animal/.

[49] Figdor C. 2024 Individuating cognitive characters: lessons from praying mantises and plants. Phil. Sci. **91**, 930–949. (10.1017/psa.2024.10)

[50] Powell R. 2023 Social norms and superorganisms. Biol. Philos. **38**, 21. (10.1007/s10539-023-09909-x)

[51] Barron AB, Klein C. 2016 What insects can tell us about the origins of consciousness. Proc. Natl Acad. Sci. USA **113**, 4900–4908. (10.1073/pnas.1520084113) 27091981 PMC4983823

[52] Brown R, Lau H, LeDoux JE. 2019 Understanding the higher-order approach to consciousness. Trends Cogn. Sci. **23**, 754–768. (10.1016/j.tics.2019.06.009) 31375408

[53] LeDoux JE. 2021 As soon as there was life, there was danger: the deep history of survival behaviours and the shallower history of consciousness. Phil. Trans. R. Soc. B **377**, 20210292. (10.1098/rstb.2021.0292) 34957848 PMC8710881

[54] Vallortigara G. 2021 The efference copy signal as a key mechanism for consciousness. Front. Syst. Neurosci. **15**, 765646. (10.3389/fnsys.2021.765646) 34899201 PMC8662721

[55] Veit W. 2023 Complexity and the evolution of consciousness. Biol. Theory **18**, 175–190. (10.1007/s13752-022-00407-z)

[56] Baars BJ. 2005 Global workspace theory of consciousness: toward a cognitive neuroscience of human experience. Prog. Brain Res. **150**, 45–53. (10.1016/S0079-6123(05)50004-9) 16186014

[57] Baars BJ, Geld N, Kozma R. 2021 Global workspace theory (GWT) and prefrontal cortex: recent developments. Front. Psychol. **12**, 749868. (10.3389/fpsyg.2021.749868) 34899489 PMC8660103

[58] Veit W. 2022 The origins of consciousness or the war of the five dimensions. Biol. Theory **17**, 276–291. (10.1007/s13752-022-00408-y)

[59] Birch J. 2024 The Edge of Sentience: Risk and Precaution in Humans, Other Animals, and AI. Oxford, UK: Oxford University Press. (10.1093/9780191966729.001.0001)

[60] Cleeremans A, Tallon-Baudry C. 2022 Consciousness matters: phenomenal experience has functional value. Neurosci. Conscious. **2022**, niac007. (10.1093/nc/niac007) 35479522 PMC9036654

[61] Panksepp J, Lane RD, Solms M, Smith R. 2017 Reconciling cognitive and affective neuroscience perspectives on the brain basis of emotional experience. Neurosci. Biobehav. Rev. **76**, 187–215. (10.1016/j.neubiorev.2016.09.010) 27640756

[62] Carranza-Pinedo V. 2024 Rethinking core affect: the role of dominance in animal behaviour and welfare research. Synthese **203**, 153. (10.1007/s11229-024-04591-2)

[63] Carruthers P. 2023 On Valence: Imperative or Representation of Value? Br. J. Philos. Sci. **74**, 533–553. (10.1086/714985)

[64] Bartal IBA. 2024 The complex affective and cognitive capacities of rats. Science **385**, 1298–1305. (10.1126/science.adq6217) 39298607

[65] Carruthers P. 2018 Valence and value. Phil. Phenomenol. Res. **97**, 658–680. (10.1111/phpr.12395)

[66] Sober E. 2015 Ockham’s razors: a user’s manual. Cambridge, UK: Cambridge University Press. (10.1017/CBO9781107705937)

[67] Mikhalevich I. 2015 Experiment and animal minds: why the choice of the null hypothesis matters. Phil. Sci. **82**, 1059–1069. (10.1086/683440)

[68] Meketa I. 2014 A critique of the principle of cognitive simplicity in comparative cognition. Biol. Phil. **29**, 731–745. (10.1007/s10539-014-9429-z)

[69] Kauppinen A. 2021 Relational imperativism about affective valence. In Oxford Studies in the Philosophy of Mind (ed. U Kriegel), pp. 341–370, vol. 1. Oxford, UK: Oxford University Press. (10.1093/oso/9780198845850.003.0012)

[70] Paul ES, Sher S, Tamietto M, Winkielman P, Mendl MT. 2020 Towards a comparative science of emotion: affect and consciousness in humans and animals. Neurosci. Biobehav. Rev. **108**, 749–770. (10.1016/j.neubiorev.2019.11.014) 31778680 PMC6966324

[71] Gillette R, Brown JW. 2015 The sea slug, Pleurobranchaea californica: A signpost species in the evolution of complex nervous systems and behavior. Integr. Comp. Biol. **55**, 1058–1069. (10.1093/icb/icv081) 26163678 PMC4836448

[72] Mahr JB, Fischer B. 2023 Internally triggered experiences of hedonic valence in nonhuman animals: cognitive and welfare considerations. Perspect. Psychol. Sci. **18**, 688–701. (10.1177/17456916221120425) 36288434

[73] Arnaud S. 2025 Unconscious emotions. Erkenntnis **90**, 285–304. (10.1007/s10670-023-00698-z)

[74] Lacewing M. 2007 Do unconscious emotions involve unconscious feelings? Philos. Psychol. **20**, 81–104. (10.1080/09515080601023402)

[75] Hatzimoysis A. 2007 The case against unconscious emotions. Analysis **67**, 292–299. (10.1093/analys/67.4.292)

[76] Deonna J, Teroni F. 2020 Affective consciousness and its role in emotion theory. In The Oxford Handbook of the Philosophy of Consciousness (ed. U Kriegel), pp. 102–123. Oxford, UK: Oxford University Press. (10.1093/oxfordhb/9780198749677.013.5)

